# Transcriptomic and presence/absence variation in the barley genome assessed from multi-tissue mRNA sequencing and their power to predict phenotypic traits

**DOI:** 10.1186/s12864-019-6174-3

**Published:** 2019-10-29

**Authors:** Marius Weisweiler, Amaury de Montaigu, David Ries, Mara Pfeifer, Benjamin Stich

**Affiliations:** 1 Institute for Quantitative Genetics and Genomics of Plants, Universitätsstraße 1, Düsseldorf, 40225 Germany; 2 Cluster of Excellence on Plant Sciences, From Complex Traits towards Synthetic Modules, Universitätsstraße 1, Düsseldorf, 40225 Germany

**Keywords:** Barley, Multi-tissue transcriptomics, Presence/absence variation, ^∗^Omic prediction, Genomic selection

## Abstract

**Background:**

Barley is the world’s fourth most cultivated cereal and is an important crop model for genetic studies. One layer of genomic information that remains poorly explored in barley is presence/absence variation (PAV), which has been suggested to contribute to phenotypic variation of agronomic importance in various crops.

**Results:**

An mRNA sequencing approach was used to study genomic PAV and transcriptomic variation in 23 spring barley inbreds. 1502 new genes identified here were physically absent from the Morex reference sequence, and 11,523 previously unannotated genes were not expressed in Morex. The procedure applied to detect expression PAV revealed that more than 50% of all genes of our data set are not expressed in all inbreds. Interestingly, expression PAV were not in strong linkage disequilibrium with neighboring sequence variants (SV), and therefore provided an additional layer of genetic information. Optimal combinations of expression PAV, SV, and gene abundance data could enhance the prediction accuracy of predicting three different agronomic traits.

**Conclusions:**

Our results highlight the advantage of mRNA sequencing for genomic prediction over other technologies, as it allows extracting multiple layers of genomic data from a single sequencing experiment. Finally, we propose low coverage mRNA sequencing based characterization of breeding material harvested as seedlings in petri dishes as a powerful and cost efficient approach to replace current single nucleotide polymorphism (SNP) based characterizations.

## Background

A priority of modern agriculture is to increase the productivity of crops to meet the demands of a growing human population. The urge of achieving significant yield gains is amplified by the current context of climate change, competition for land, and limited natural resources [1]. Plant genetics and breeding are considered among the disciplines that have the highest potential to tackle this challenge. One of the major approaches used in plant breeding to increment yield gains is to exploit the natural genetic variation present in the crop species’ gene pool. Barley was domesticated more than 10,000 years ago in the fertile crescent [2]. Its cultivation area has progressively expanded to a wide range of latitudes, and it is now the fourth most important cereal in the world [3]. Barley has also become an important model cereal species for research, partly because its tolerance to stress surpasses that of other major crops including wheat and rice [4]. Moreover, the diploid genome of barley facilitates genetics studies.

To exploit the natural genetic variation present in the gene pool of barley, genomic tools such as single nucleotide polymorphism (SNP) arrays have been developed [5]. The availability of a reference genome sequence facilitates the use of next generation sequencing technologies for the discovery of novel sequence variants [6]. This allowed e.g. to characterize most of the barley accessions of the German ex situ genebank using a genotyping by sequencing approach [7].

Genome wide quantification of gene expression has also been accessible in barley since many years through the development of gene expression arrays [8]. This technology allowed addressing how the barley transcriptome varied between tissues [9], and how it responded to pathogens and to environmental cues such as vernalization and heat [10–13]. eQTL studies with these arrays further revealed a complex pattern of genome-wide regulation of barley genes [14], and described how limited pleiotropy acted on gene expression in a tissue dependent manner [15]. With the release of a high quality reference sequence, resequencing technologies are successfully providing novel information on the barley genome and transcriptome that had remained inaccessible [16–18].

It is now accepted that a significant proportion of the genes of plant genomes are not expressed (expression presence/absence variation; ePAV) or are even completely absent (genomic PAV; gPAV) in subsets of genotypes, and make up what is known as the dispensable transcriptome or genome [19–21]. The occurrence of PAV in crops has extensively been reported for maize and rice [19–25]. However, up to now, little information is available concerning the extent and distribution of PAV in the barley genome [18, 26].

Prediction of phenotypic variation in the context of genomic selection, which is nowadays an essential component of plant breeding programs, is performed based on SNP genotyping profiles. Previous studies on the use of metabolome and lipidome variation to predict phenotypic traits of maize revealed high but lower prediction accuracies compared to SNP information [27, 28]. Only the use of microarray based transcriptome information for prediction of phenotypic traits in maize resulted for a subset of the traits in increased prediction accuracies especially when combined with SNP genotyping information [29]. However, the transcriptomic characterization of genotypes by mRNA sequencing has the advantage that also SNP information can be extracted from such a data set. In addition, the cost of characterizing genetic material by mRNA sequencing can be influenced by modifying the sequencing depth. Despite these advantages, no earlier study examined the prediction accuracies of predictors extracted from mRNA sequencing data sets. Furthermore, an evaluation of the prediction accuracy of PAV has to our knowledge not yet been performed, despite that single PAV have been shown to contribute to phenotypic variation of selected traits in various crops (for review see Gabur et al. [30]).

In this study, we explored the genomic and transcriptomic landscape of 23 spring barley landraces and cultivars which were selected based on their genetic and phenotypic diversity as parents of a joint linkage and association mapping population. The objectives of our study were to (i) characterize genomic and transcriptomic variation in the barley genome using multi-tissue mRNA sequencing, (ii) assess the proportion of ePAV that are due to gPAV, (iii) examine how accurately the different layers of genomic and transcriptomic variation predict phenotypic variation of various agronomic traits.

## Results

To study genomic diversity in spring barley inbreds, we first selected 23 inbreds from a panel of 224 representing a broad range of origins [31] (Additional file 1: Table S1). mRNA was extracted from seedlings and leaves of all of these 23 inbreds, and from apex of a subset of six inbreds (Additional file 1: Table S1). Gene expression was determined for each individual sample by sequencing the mRNA. Out of the 73,187 expressed genes across seedlings, leaves, and apex samples, 11,523 genes mapped to regions of the Morex reference genome where no gene had previously been annotated (Additional file 1: Figure S1). We considered a gene as newly annotated gene if it was detected in at least two samples. A total of 3,482 genes mapped to the unknown chromosome of the Morex reference sequence, where 581 of these were newly annotated genes. The average length of the newly annotated genes was 5,470 bp.

**Fig. 1:**
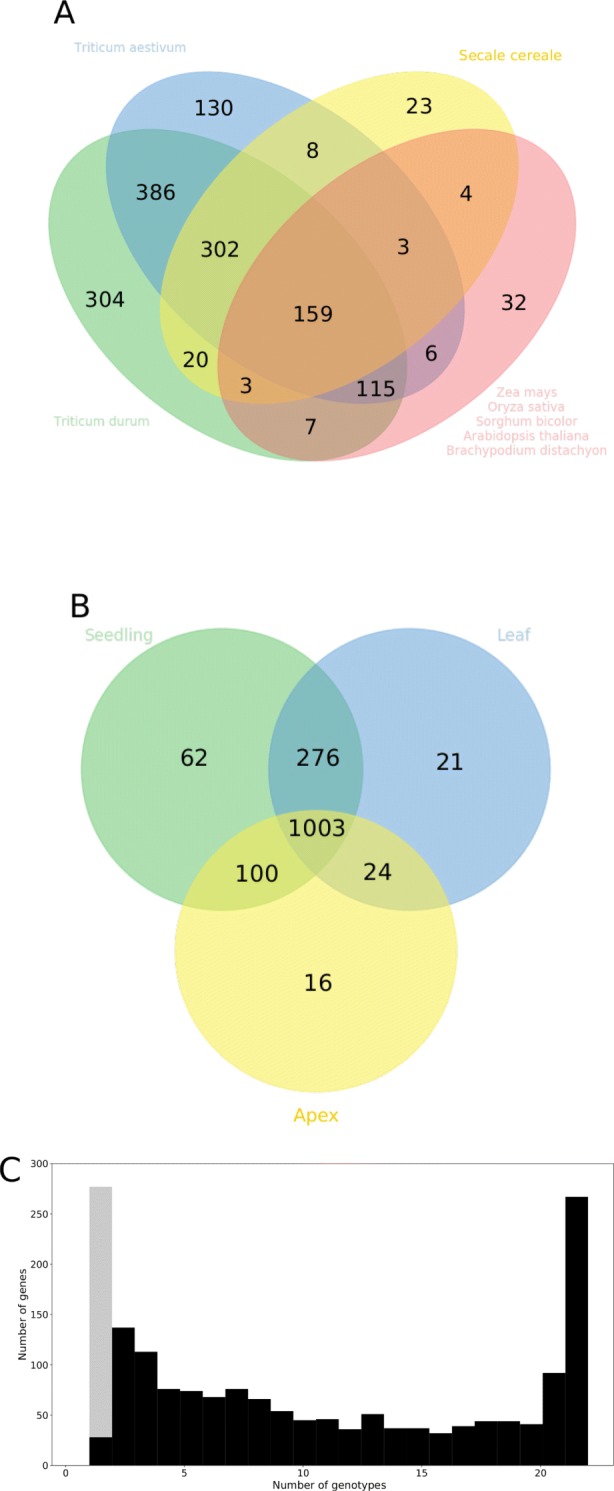
Characterization of the contigs established by a *de novo* transcriptome assembly of unmapped reads across all 23 inbreds. **a** Number of newly identified genes which had based on BLASTn searches homology to at least one of eight different plant species. **b** Expression of 1,502 newly identified genes in the three different tissues. **c** Number of inbred lines in which the contigs of the *de novo* transcriptome assembly were expressed. Gray bar shows the contigs detected in only one sample. The 1502 genes, which were expressed in at least two samples, were marked in black and were designated as newly identified genes

We additionally identified 1,502 new contigs, with an average gene length of 494 bp, that did not map to any of the seven barley chromosomes. These contigs were designated in the following as newly identified genes, although a portion of these contigs might not actually be protein coding genes, if they were expressed in at least two samples, and if they showed homology to at least one gene of one out of eight plant species. In total, 96% of the homologous genes were found in other cereals of the *Triticacea* tribe but not in more distantly related species (Fig. 1A), indicating that they were not conserved across the plant kingdom but might fulfill functions specific to barley and closely related species. In addition, only 280 of the newly identified genes had an unknown gene annotation compared to the eight plant species. Altogether, 67% of the newly identified genes were expressed in all three tissues (Fig. 1B), making it unlikely that they are due to technical artifacts. We next tested if the newly identified genes were found predominantly in isolated inbreds, or if their presence was common across our set of spring barley accessions. This analysis revealed that about 25% of newly identified genes were expressed in isolated inbreds, and another 25% in all inbreds (except Morex, Fig. 1C).

**Fig. 2:**
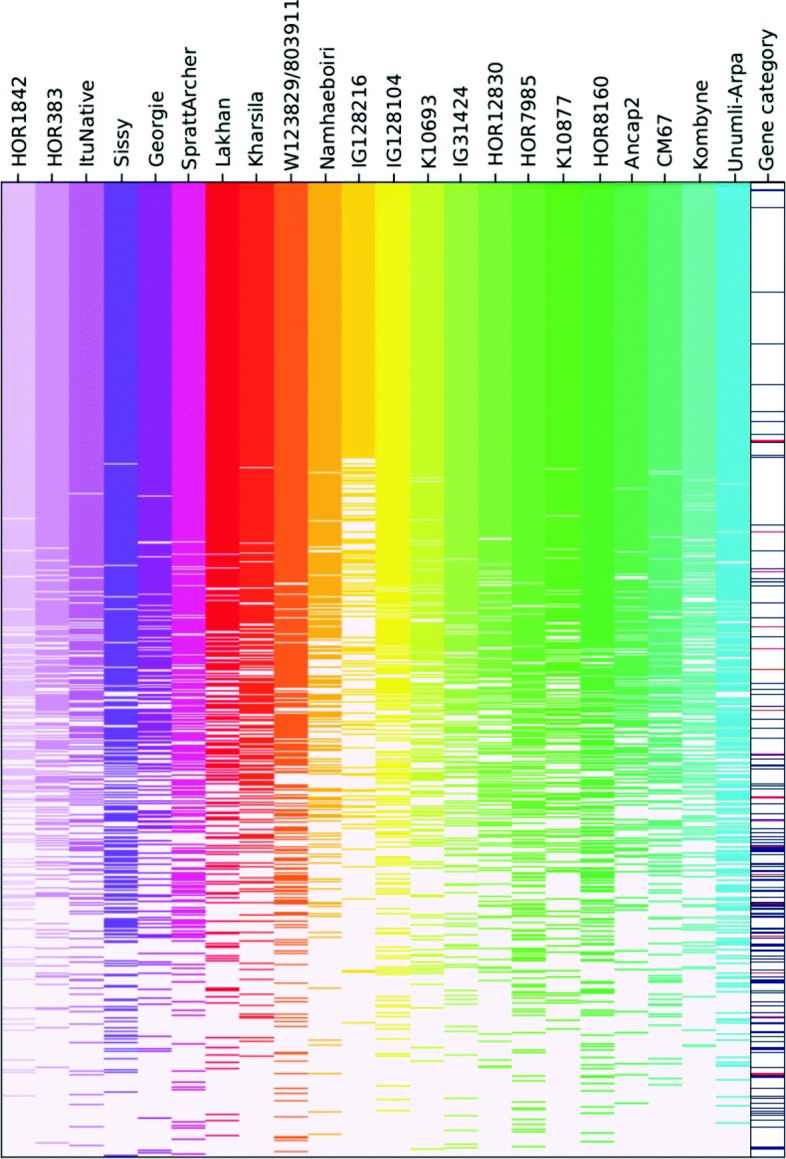
Gene expression of all inbreds. Presence and absence of the 73,187 genes across all inbreds. Genes were sorted according to their presence across all inbreds (top to bottom). Presence of a gene is highlighted as colored and absence as white bar. The last column illustrates the three different gene categories as white (IBSC), blue (newly annotated), and red (newly identified) bars

**Fig. 3:**
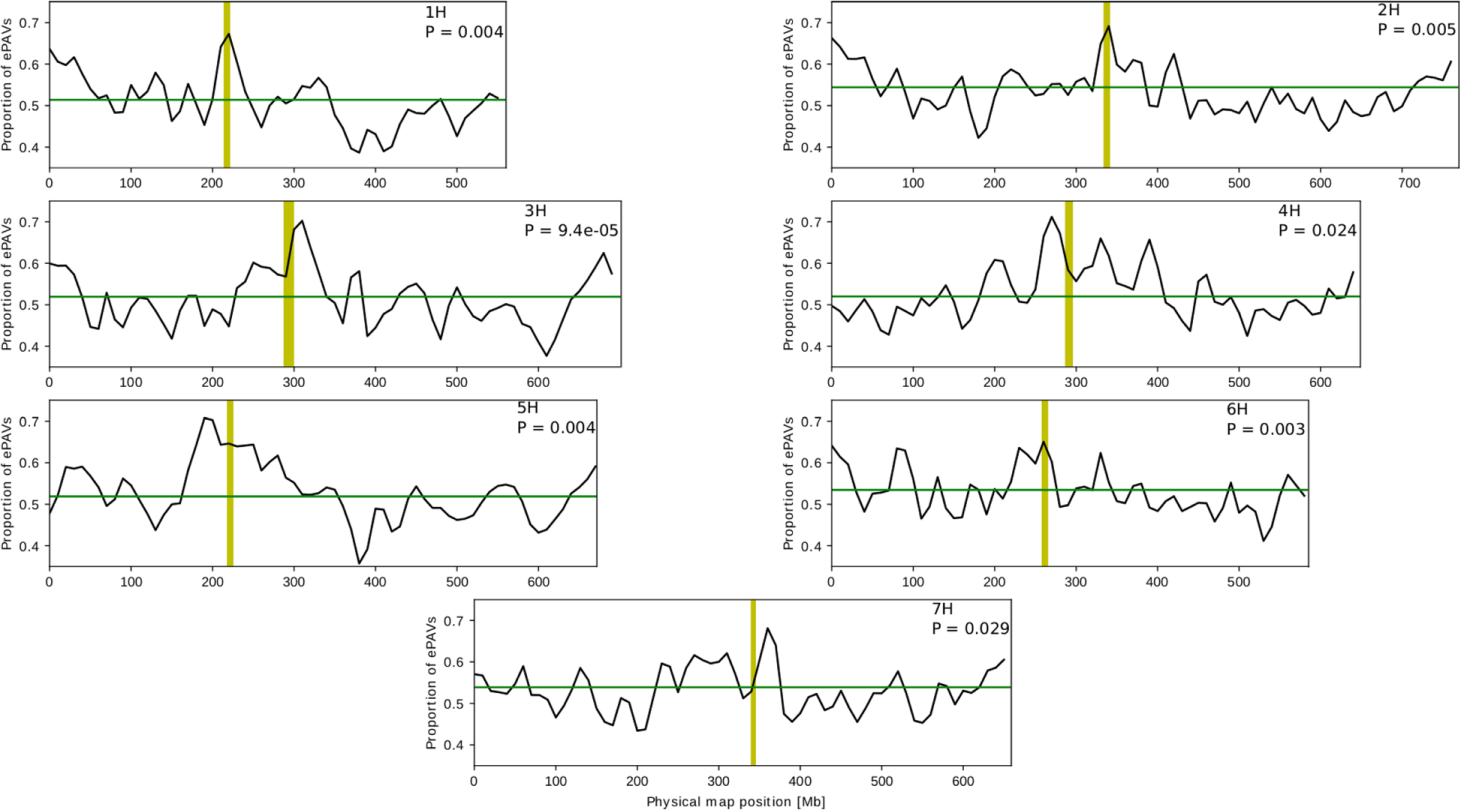
Distribution of expression presence/absence variation (ePAV) across the physical map of the barley chromosomes. Proportion of ePAV among all genes within sliding windows of 20 Mb is given on the y-axis. The green line shows the average proportion of ePAV among all genes of a chromosome and the P value indicates, if the distribution deviates significantly from an uniform distribution. The yellow line illustrates the centromeric region

The high number of genes absent in Morex inspired us to systematically explore ePAV among the 23 inbreds. ePAV were defined as genes whose expression was detected/not detected in at least two inbreds. A total of 38,810 barley genes were detected as ePAV, of which 28,340 had previously been annotated in the reference genome (Additional file 1: Table S2). ePAV were enriched in genes implicated in very diverse biological processes (Additional file 1: Table S3). The average length of ePAV (4162 bp) was significantly shorter than that of non-ePAV genes (9458 bp). In contrast, the average coding sequence length of ePAV (411 bp) was longer than that of non-ePAV genes (282 bp). Non-ePAV genes only rarely corresponded to newly identified genes or newly annotated genes (Fig. 2), and in fact, 80.6% of the newly annotated genes and 78.8% of the newly identified genes were also detected as ePAV (Additional file 1: Table S2). ePAV were significantly (P <0.05) unevenly distributed along the chromosomes, with the highest frequency of occurrence close to the centromers (Fig. 3).

The robustness of our ePAV detection procedure was evaluated using a resampling simulation. In 50 replications, 20% of the gene length of each gene was used for transcript calling and ePAV detection. Across the 50 replications, the average number of genes as well as the number of detected ePAV was about 67,000 and 35,400, respectively, only slightly lower than the 73,187 and 38,810 detected when considering the entire gene length (Additional file 1: Table S2).

Information on the proportion of ePAV that are due to gPAV is generally scarce. In order to estimate it, we used SNP genotyping profiles of segregating populations. The 23 inbreds had previously been crossed following a double round robin design [32] to generate a joint linkage and association mapping population. SNP genotyping profiles obtained with a 50K SNP array were available for these 45 populations. We searched for SNP for which missing data were segregating as a monogenic character, and used this pattern to assign presence/absence calls to the parental inbreds. Using such SNP located within genes, which we refer to as gPAV-SNP, we calculated the proportion of gPAV-SNP that were also detected by our procedure as ePAV, and considered this value as an estimation of the power to detect gPAV by our ePAV detection procedure (Additional file 1: Figure S2).

Based on the criterion that a gene is considered an ePAV if it has a present and an absent call in at least two inbreds, the power of gPAV detection was 34.6% (Table 1). This means that out of all gPAV, which we detected based on gPAV-SNP from the SNP array data, we identified 34.6% of it as ePAV in our mRNA sequencing data. It could be possible that parts of genes are still expressed even though a small fragment of their sequence was deleted. In this case, the genes would have a presence call though the region around the gPAV-SNP were not covered by reads. For this reason, we also calculated the power of our procedure when detecting ePAV exclusively based on the 10bp sequence window surrounding the gPAV-SNP instead of using FPKM-values for the entire gene sequences. In this case, the power increased to 53%. The empirical type I error rate, defined as the proportion of ePAV that are not gPAV, was about 90%. Finally, the similarity between presence/absence patterns in the 23 inbreds of ePAV and gPAV-SNP was very high, ranging from 70% to 90%.

**Table 1:** Detection procedure of presence/absence variation

		Expression				Expression		
		of gene				of ±5bp genic SNP		
Tissue	*t*	1- *β*^∗^	*α* ^∗^	*o*		1- *β*^∗^	*α* ^∗^	*o*
Leaf&Seedling&Apex	1	45.0	88.8	88.6		64.2	90.8	85.3
	2	34.6	87.5	81.8		53.0	90.1	81.8
	3	30.1	86.7	79.5		44.8	90.0	79.5
	4	25.1	87.0	77.3		38.4	89.8	77.3
	5	21.4	86.5	77.3		32.7	89.7	77.3
Leaf&Seedling	2	35.2	87.5	81.8		52.4	90.3	79.5
Leaf	2	34.0	89.0	73.8		45.9	91.2	72.2
Seedling	2	28.1	86.7	76.7		45.9	90.5	76.2

Statistical power (1−*β*^∗^) and the empirical type I error rate (*α*^∗^) to detect genomic presence/absence variation (gPAV) by expression PAV (ePAV), where *t* is the minimum number of inbreds that must have a present and absent call for a gene, *o* the percentage of common presence/absence values across all inbreds between ePAV and the genic PAV-SNP. We considered two scenarios: (i) the expression across the entire gene or (ii) the expression determined in a 10 bp window around the genic SNP was used to determine ePAV.

We were interested in knowing how independent the detected ePAV are from the local genomic pattern. First, the mRNA sequencing data was used to call sequence variants (SV) within exon sequences. A total of 133,566 SV were detected. We then determined the extent of linkage disequilibrium (LD) between each ePAV and neighboring SV located within 100 kb. Only 17.5% of all ePAV have at least one SV within 100 kb that has an *r* ^2^≥ 0.4 (Table 2). This figure is even lower than for SV that are located outside the 100 kb window. In contrast, more than 85% of SV that are neighboring an ePAV show an *r* ^2^≥ 0.4 with another SV within 100 kb. Therefore, ePAV provide an additional layer of genetic information compared to SV. This idea was confirmed by comparing principal component analyses (PCA) performed based on SV and ePAV. Both PCA revealed the existence of two clusters of inbreds defined by the row type of the inbreds (Additional file 1: Figure S3). Principal components 1 from both PCAs were significantly correlated with each other (*r* ^2^= 0.4928709, p = 0.0002706), and a similar result was observed for principal components 2 (*r* ^2^= 0.3980411, p = 0.001643). However, these analyses also reveal that the relationship of the inbreds within clusters differs between the two sources of molecular variation. A similar trend was observed when comparing the transcriptomic variation (T) with that of ePAV and SV. Mantel tests of distance matrices calculated from T, SV, and ePAV data indicated only significant correlations between the seedling transcriptome and SV (r = 0.2581, p = 0.03969).

**Table 2:** Linkage disequilibrium between expression presence/absence variation (ePAV) and sequence variants (SV)

*r* ^2^	[1.0,0.8]	(0.8,0.6]	(0.6,0.4]	(0.4,0.2]	(0.2,0]
	Percentage of *r*$^{2}_{max}$ between ePAV and SV				
linked	0.0	0.0	17.5	49.9	31.1
unlinked	0.0	0.1	23.4	54.0	22.1
	Percentage of *r*$^{2}_{max}$ between closest SV beside ePAV and SV				
linked	0.0	34.1	52.9	12.9	0.0
unlinked	0.0	21.1	52.7	25.2	0.0

Percentage of expression presence/absence variant or its closest neighboring sequence variant that show a maximum linkage disequilibrium estimate *r*$^{2}_{max}$ to the SV 100 kb up and downstreams of it (linked) or outside that interval (unlinked) for five *r*^2^ classes.

Therefore, we examined the prediction accuracy that can be obtained when predicting the traits leaf angle, heading date, and plant height, for which h^2^ values between 0.69 and 0.76 were observed. In order to obtain unbiased estimates of the prediction accuracy, we randomly subdivided in 1000 cross-validation runs the 23 inbreds in training and validation set. Prediction accuracies of SV, T, and ePAV were compared to the prediction accuracy of the SNParray data set that we used as the baseline predictor. The median prediction accuracy across 1000 cross-validation runs observed for the SNParray data set ranged from -0.49 for heading date to 0.70 for leaf angle (Additional file 1: Figure S4). We observed across the three traits a slightly higher prediction accuracy for the SV extracted from the mRNA sequencing data set compared to the SNParray. An even higher prediction accuracy was observed when using ePAV as predictor. The seedling transcriptome (T _*s*_) resulted across the three traits in the highest median of prediction accuracy of all the examined single predictors.

**Fig. 4:**
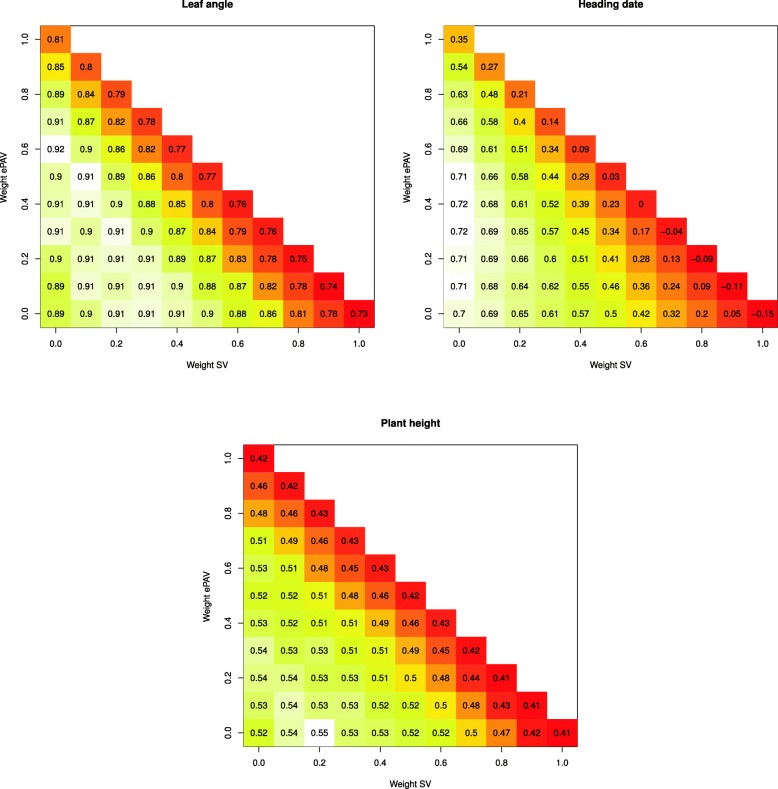
Prediction accuracy for three different traits. Prediction accuracy for the barley inbreds for leaf angle, heading date, and plant height, for 66 cases which differ in their weights for the predictors sequence variants (SV), expression presence/absence variation (ePAV), and gene expression in seedlings (T _*s*_). Their corresponding relationship matrices were joined with weights varying from 0 to 1 in increments of 0.1. Weights for SV and ePAV are shown at the respective scales; weights for gene expression are = 1 - weight of SV - weight of ePAV. Plotted values represent medians of prediction accuracy across 1,000 cross-validation runs. Heat color schemes differ for the three traits ranging from white, indicating the respective highest value, to red for the respective lowest value

**Fig. 5:**
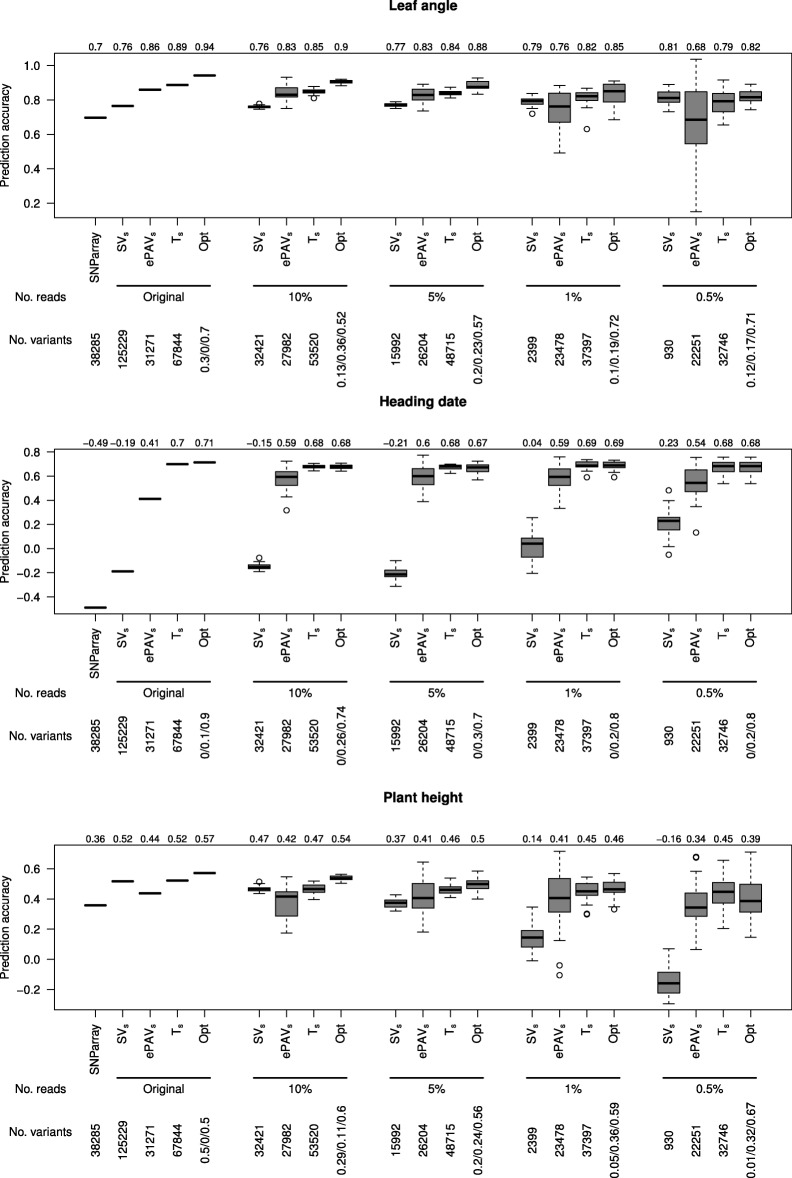
Prediction accuracy for reduced number of mRNA sequencing reads. Prediction accuracy for the barley inbreds for leaf angle, heading date, and plant height of single predictors as well as the optimal combination identified in a grid search (Opt) using the original number of reads of the seedling sample as well as using data sets for which the number of reads was randomly reduced to 10, 5, 1, and 0.5% of the original number of reads per seedling sample. The number of variants gives the mean number of features available for predictions in each scenario or for the combined predictors the weight of sequence variants (SV _*s*_)/expression presence/absence variation (ePAV _*s*_)/gene expression (T _*s*_) resulting in the highest prediction accuracy. The median prediction accuracy is given above each column

We also evaluated the pairwise combinations of single predictors and observed for all traits an increase of the prediction accuracy compared to using T _*s*_. Therefore, a grid search in which the relative weights of the relationship matrices of two or three predictors varied in increments of 0.1 prior to summing them up, was used to identify those combinations of SV, ePAV, and T _*s*_ that resulted in the highest prediction accuracies. For all three traits, the highest median of the prediction accuracy was observed when using more than one predictor (Fig. 4). Furthermore, a common trend was that the optimal weight of T _*s*_, i.e. the weight that maximizes the prediction accuracy, was at least 40%, whereas the optimal weight of ePAV and SV differed among traits. We examined the prediction accuracy of single predictors as well as optimal combinations of predictors determined from seedling samples sequenced at different depths. Across all traits, we observed that the prediction accuracies decreased for decreasing sequencing depth (Fig. 5). However, the extent of reduction differed between the different predictors and was most pronounced for the SV. The prediction accuracies observed for the optimal combinations of predictors reduced for the three traits only slightly with decreasing sequencing depth. Even with a sequencing depth that corresponds to 0.5% of that of our study, prediction accuracies higher than that of the prediction with the SNParray data set were obtained. However, the variability of the prediction accuracy across the different runs of the resampling simulations increases with a reduced sequencing depth.

## Discussion

### Transcriptomic variation in barley

Across the 23 inbreds of our study, we have identified 11,523 previously unannotated genes that are not expressed in Morex. Furthermore, we assembled 1,502 newly identified genes that are physically absent from the Morex genome, and are therefore part of barley’s dispensable genome. Both numbers are in the range of what was previously reported for barley [17, 18] as well as maize [20, 21]. These genes were added to the standard International Barley Sequencing Consortium (IBSC) gene list and the resulting list was the basis for all following analyses.

Across the three tissues, we observed that about 53% of the total number of genes were detected as ePAV (Additional file 1: Table S2). Despite our lower sample size and the use of three tissues, which both reduce the number of detected ePAV, this figure is considerably higher than the maximum 30% that were observed for maize [20, 21]. Because the proportion of ePAV detected here is consistent with other studies in barley [18], the discrepancy of our results and those from maize possibly imply that the proportion of ePAV within a pan-transcriptome is species specific. Due to the unique domestication histories of every crop, selective pressures may have acted differently on dispensable genomes and transcriptomes, especially in cases where PAV variation provided benefits [30]. It is also possible that large genomes rich in repetitive sequences and transposable elements such as the genome of barley contain higher numbers of non-essential genes, whose loss of function has no major impact on plant physiology and can be tolerated by the organism.

We observed that ePAV genes were significantly shorter than an average barley gene. A similar observation was made by Bush et al. [33] in *Arabidopsis thaliana*. Tan et al. [34] described the same trend, although in this case the authors reported variations in gene size (200 bp) that were much smaller than the variations between PAV and non-PAV genes detected by us (5 kb) and by others (1.5 kb) [33]. Another feature that we observed for ePAV genes is that the likelihood that a gene is an ePAV is significantly (P <0.05) unequally distributed across the genome. We observed the highest proportion of ePAV among the present genes in centromeric regions (Fig. 3). This might be explained thereby that selection is less efficient in lowly recombining regions of the chromosome compared to highly recombining pericentromeric regions to purge presence/absence variation that was created by evolutionary processes during plant polyploidization and speciation [30]. gPAV and ePAV were shown to be enriched in genes implicated in disease resistance and stress responses [18, 26, 34–36]. However, the gene ontology (GO) term analysis of the ePAV detected here did not reveal an enrichment of genes implicated in these processes, neither in any other process that could be related to crop performance or adaptation (Additional file 1: Table S3). Further research is required to understand the reason for this difference.

### Detecting gPAV by mRNA sequencing

The absence of a gene in a genotype, i.e. the gene is an ePAV, has two possible causes: either the corresponding gene is transcriptionally inactive or it is physically absent from the genome, i.e. it is a gPAV. We were interested in estimating the proportion of ePAV that are due to gPAV. In order to do so, we detected in analogy to Gabur et al. [37] gPAV from the segregation of missing data in biparental populations. This allowed us to estimate that by characterizing the expression of genes in one tissue, we are able to detect about 30% of the existing gPAV. However, a SNP for which a systematic segregation of missing data was observed and that was designated as a gPAV does not necessarily mean that the entire gene in which the SNP is located is missing and therefore not expressed. Instead, the gPAV can be also caused by partial insertion/deletions of the corresponding gene. Therefore, we also examined the power to detect gPAV (1- *β*^∗^) for a scenario in which only the gene expression in a window of 10 bp around the SNP was considered. In this case of using a single tissue to detect gPAV, the proportion of gPAV that are detected by our ePAV procedure increases to about 46% (Table 1). These findings indicated that our ePAV detection procedure is therefore powerful in detecting gPAV. Furthermore, we observed that 1- *β*^∗^ can be increased even more, if multiple tissues were studied. However, this increase was not of such a size that it justifies the additional resources.

In addition to estimating the power of gPAV detection 1- *β*^∗^, we were also interested in estimating the proportion of ePAV that are not due to gPAV *α*^∗^. *α*^∗^ was approximately 90% in our data set (Table 1), meaning that 10% of the ePAV are caused by the physical absence of the gene and not by impairment of its transcription. This proportion is considerably higher than the 1% reported in maize [21]. An explanation for this finding might be that deletions of entire genes and perhaps of even larger segments of DNA are better tolerated in barley than maize. There is not enough available information on structural variation in the barley genome to be able to compare deletion sizes and frequencies between barley and maize, but it is possible that the presence of long stretches of repetitive elements in barley may have an influence on this process. Another explanation could be the differences in the methodologies between both studies. Jin et al. [21] had used resequencing data to detect gPAV, where our procedure based on patterns of missing data in segregating populations could be more sensitive.

### Number of dispensable genes in the barley genome

We can estimate from the above described estimates of 1- *β*^∗^ and *α*^∗^ that about 10% of the about 38,000 ePAV, i.e. 3,800, are gPAV. With a power 1- *β*^∗^ of about 50% of our ePAV procedure to detect gPAV, the total number of gPAV is expected to be around 7,600 for barley. Therefore, our results suggest that more than 10% of the barley genes show PAV on a genomic level. This figure is similar to what was observed in the analysis of 80 *Arabidopsis* accessions (9%) [34], but was higher compared to other cereal species. Springer et al. [23] estimated that 8.6% of all genes were gPAV in maize. However, their set of analyzed genotypes included, in addition to 19 maize inbreds, 14 wild ancestors, and therefore encompassed a higher genetic diversity compared to our study. As this increases the proportion of detected gPAV, it suggests that cultivated maize might have a lower gPAV diversity than barley. Consistently, a study in rice also including cultivars and wild relatives estimated to 10% the proportion of gPAV [35], suggesting that rice may also have a lower gPAV diversity than barley. But beyond the proportion of gPAV that a species may contain comes the question of what impact on plant physiology and performance this variation might have. Despite clear examples of gPAV controlling agronomic traits in different species, it has been proposed that most gPAV are not essential and are recent additions to plant genomes [33]. Further data will be necessary to elucidate why enrichment of functional categories occur in certain data sets and not in others, and whether PAV variation has played a more important role in the evolution of crops compared to non-crop species as studies so far seem to suggest [30, 33].

### Genomic and transcriptomic prediction

Genomic prediction is becoming a standard tool for plant breeders to increase the gain of selection [38]. The current implementation of genomic selection is mainly based on the use of SNP markers assessed by SNP arrays or genotyping by sequencing methods (for review see Crossa et al. [39]). However, we have observed considerable variation of T as well as ePAV and, very importantly, this variation was largely independent from the variation explained by neighboring SNP (Table 2). Therefore, the accuracy of T and ePAV to predict phenotypic traits was assessed.

We have observed that for all three examined traits both types of information that were extracted from the mRNA sequencing data set, the SV as well as the ePAV, resulted in higher prediction accuracy when using GBLUP compared to the classically used SNP data generated with a 50K SNParray (Additional file 1: Figure S4). For SV, that might be explained by the higher number of features compared to the SNParray information, which in turn increases the extent of LD between the SNP and the QTL [40]. However, for the ePAV this was not the case. Instead, the superiority of the ePAV information compared to the SNParray for the prediction of phenotypic traits might be due to that ePAV are only caused to 10% by gPAV but cover also gene expression differences. The transcriptome T is expected to incorporate gene expression and physiological epistasis [41] and therefore has a considerably higher prediction accuracy compared to SV or SNParray (Additional file 1: Figure S4), even when modelling statistical epistasis.

However, we also observed differences in the prediction accuracy of T depending on the tissue that was used for mRNA extraction. The prediction accuracies were on average across the three examined traits considerably higher for T _*s*_ compared to T _*l*_ data set. This finding might be explained either by the fact that the number of cell types that were included for mRNA extraction were more diverse for the former than the latter and thereby increases the number of features from 60,888 to 67,844. Another non mutually exclusive possible explanation is that the time of heterogenous environmental factors to influence the genotypes was lower for the seedling samples compared to the leaf samples. And in the set-up used in our study of unreplicated plants for sample collection such heterogenous environmental factors cause together with genotype*environment interaction a reduction of the precision of the measurement of the predictor. This in turn is expected to reduce the prediction accuracy. Our finding indicated that the transcriptome of seedlings grown on filter paper is a good proxy of the gene activity for a broad range of developmental stage of plants grown in a diverse set of environments.

Schrag et al. [29] derived from a comparison of pairs of single predictors with their combinations the following two conclusions. First, combining the best single predictor for a certain trait with another predictor did not improve predictions and in some cases rather impaired predictive ability. Second, combinations that did not comprise the best single predictor tended to be superior to both components individually. Both of them were not in agreement with our findings. Instead, we have observed a complementarity between the best single predictor T _*s*_ and SV and even between T _*s*_ and ePAV (Additional file 1: Figure S4).

Therefore, a grid search was used to identify those combinations of SV, ePAV, and T _*s*_ that maximize the prediction accuracy. For all three traits, the highest median of the prediction accuracy was observed when using more than one predictor (Fig. 4). In contrast to the results of Schrag et al. [29] and Xu et al. [35], we have observed rather small differences between the optimal weight of the three predictors across the three examined traits, despite that these were assessed at completely different developmental stages. The likely explanation for this difference is that, in contrast to Schrag et al. [29] and Xu et al. [35], we focused on genetic and transcriptional predictors and did not include features derived from metabolome analyses, which represent a completely different level of information.

### Application in breeding

In the above described grid search, the SV and ePAV data sets were extracted from the mRNA sequencing data of multiple tissues. A cost efficient integration of our approach in practical breeding programs would require that all data sets are extracted from the sequence experiment of one tissue. Due to the above described quantitative genetic advantage of the seedling sample but also the logistical advantages of using seedling samples that are generated on filter paper in petri dishes: they require a much lower amount of space, personnel and material resources, allow a season independent cultivation, as well as can be generated faster as the turn over time is shorter, they were studied in detail.

The prediction accuracy of the original sequencing depth was not influenced by predicting the phenotypic traits from SV and ePAV features extracted from the seedling sample instead from the three tissues. This can be explained by the fact that the SV _*s*_ can be adequately assessed also with one tissue and differences between ePAV _*s*_ and ePAV are compensated for by T _*s*_. The prediction accuracy observed for this scenario is considerably higher compared to using the SNParray information. However, the cost of genotyping one sample with the barley 50K SNParray is with about 50 Euro [42] also less than the mRNA sequencing analysis. When generating it newly with latest protocols and sequencing chemistry one could expect that the mRNA sequencing of one sample would cost about 2 Euro for the mRNA library preparation [43] as well as 60 Euro for 20 million 2x150 bp reads. In addition, breeding companies use for their routine genotyping in many cases smaller SNP arrays than the one used in our study. This would reduce the costs even more, but will decrease the prediction accuracy, especially, if diverse genetic material is used [44] as in our study. Therefore, we performed downsampling simulations to examine the reduction of the prediction accuracy if the sequencing depth is reduced.

The prediction accuracies observed for the optimal combinations of single predictors reduced for the three traits only slightly with a decreasing sequencing depth. The main limitation to reducing the sequencing depth to values below 1% of that of our study, i.e. about 2x10^5^ 2x150 bp reads, is not the reduction of the median of the prediction accuracy but the increasing standard deviation (Fig. 5). This increase is caused by the increasing sampling variance of the low depth sequencing. However, our results indicate that down to 5% of our data set, i.e. about 1x10^6^ 2x150 bp reads, the obtained prediction accuracy was in more than 95% of the resampling runs higher than that obtained with the SNParray data set. Such a transcriptomic characterization would cost about 5 Euro and is therefore also less expensive than current GBS approaches with the advantage of higher prediction accuracies. Therefore, we consider mRNA sequencing based characterizations of breeding material harvested as seedlings in petri dishes as a powerful and cost efficient approach to replace current SNP based characterization. For species that are bred in breeding categories other than inbred lines, the phenotypic evaluation is even more expensive [45] than for species bred as inbred lines. Therefore, an approach as suggested above will increase the gain of selection for such species even more, as the cost advantage is higher than in species bred as inbred lines.

### Conclusion

We have used mRNA sequencing as an approach to explore the dispensable genome and transcriptome of barley in 23 spring barley inbreds, and estimate that 53% of genes are ePAV. Our analyses suggest that about 10% of ePAV in barley are due to the physical absence of a gene in an inbred (gPAV). We have observed that the omic variation that was extracted from the mRNA sequencing data set, the sequence variants (SV), the ePAV, as well as the transcriptome (T) resulted individually in higher prediction accuracies compared to the classically used SNParray data set. This superiority was even more pronounced when using optimal combinations of SV, ePAV, and T to predict phenotypic traits. Finally our results suggest that low coverage mRNA sequencing based characterization of breeding material harvested as seedlings in petri dishes is a powerful and cost efficient approach to replace current SNP based characterization.

## Methods

### Plant material

Our analyses were based 23 spring barley inbreds that were selected out of a worldwide collection of 224 inbreds [31] (Additional file 1: Table S1) using the MSTRAT algorithm [46]. For these inbreds, the maximal combined genotypic and phenotypic richness index was observed. Seeds of the 23 spring barley inbreds were sown in controlled greenhouse conditions with 16 hours light and eight hours dark at 22 ^∘^C. A fragment of the youngest fully developed leaf from two different plants was collected for each inbred. The collection of all samples was done within one hour to minimize the variation due to circadian rhythms. For a total of six inbreds, apices were harvested at stage 47 of the Zadoks scale [47]. Young seedlings were harvested in an independent experiment. Seeds were surface sterilized with 1% bleach and rinsed with sterile water. Eight seeds per inbred were placed between two layers of sterile filter paper soaked with 5 mL of sterile water. The petri dishes were placed in the greenhouse with the above described environmental conditions. Five days after germination, two seedlings were sampled for each inbred. All collected samples were immediately flash frozen in liquid nitrogen. The above described experiments were performed in accordance to the experimental design of related studies [20, 21] with one biological replicate only, as the replication of alleles is provided among genotypes.

For the assessment of phenotypic traits under field conditions, the 23 spring barley inbreds were planted as replicated check genotypes in an experiment with other entries which was layed out as an augmented row column design. This experiment was performed in three environments (Cologne 2017 and 2018 and Mechernich 2018) as single row plots with 10 plants/plot as well as in a fourth environment (Quedlinburg 2018) as double row plots with 40 plants/plot. At each of the four agro-ecologically diverse environments in Germany, the 23 barley inbreds were replicated 21, 20, 19, and 19 times, respectively. For each experimental plot, three traits were assessed. The leaf angle of about four weeks old plants was scored on a scale from 1 to 9, where 1 indicates erect leaves and 9 prostrate leaves. The heading date was assessed as number of days after planting. Furthermore, the plant height in cm was assessed after heading.

### SNP genotyping and quantification of gene expression

An Illumina 50K barley SNP array [5] was used to genotype the 23 inbreds. This data set is designated in the following as SNParray. The same array was used to genotype between 35 and 146 F5 progenies of 45 segregation populations which were derived from double-chain crosses [32] of the 23 inbreds (Casale et al. in preparation).

mRNA was extracted from leaf, seedling, and apex samples (cf. Digel et al. [16]). A total of 52 polyA enriched mRNA libraries were prepared. The 150 bp paired-end Illumina sequencing libraries with individually barcoded samples were sequenced on an Illumina HiSeq2000 sequencer (Illumina, Inc., San Diego, CA USA). Reads were trimmed using trim_galore and then mapped against the unmasked barley reference sequence [6] using HISAT2 [48]. Trinity was used to perform a *de novo* assembly of the unmapped reads of all inbreds [49]. The assembled contigs that were expressed in at least two tissue samples were BLASTn-searched against a human and viral database, to exclude contigs that are due to contaminations (e-value ≤1e-5, identity ≥95.0%). Then, the contigs were searched against a barley database to remove, based on the same thresholds, genes which are too similar compared to barley reference genes. All contigs that had a homology (e-value ≤1e-5, identity ≥98.0%) to an annotated protein in at least one of the species *Arabidopsis thaliana*, *Brachypodium distachyon*, *Sorghum bicolor*, *Zea mays*, *Oryza sativa*, *Triticum aevisticum*, *Triticum dicoccum*, and *Secale cereale* were retained. The contig with the maximum coverage was chosen as representative contig for each gene [6]. These contigs were designated as newly identified genes.

Transcript calling was performed with StringTie [50] using a gene annotation file that comprised low and high confidence genes of transcripts defined in the barley reference genome [6] and the newly identified genes of the *de novo* assembly.Genes which mapped to the reference sequence and were expressed in at least two samples, but which were not available in the IBSC-reference annotation file were designated in the following as newly annotated genes. The gene expression quantified as fragments per kilobase of exon model per million fragments mapped (FPKM) is designated in the following as T, where the indexes *l, s, a* were used to separate the tissues leaf, seedling, and apex.

### Identification of ePAV

For each tissue, a presence call was made for each inbred-gene combination in the matrix of presence/absence calls, if T >0 and an absence call if T = 0. No call was made for the inbreds with 0 <*T*<10% of the maximum value of T for a gene-tissue combination (cf. Jin et al. [21]). Tissue specific ePAV calls were combined to an across tissue ePAV call as follows: If the presence/absence call made for all tissues of one inbred-gene combination was identical, this call was kept. For all inbred-gene combinations with a presence call for at least one tissue, a presence call was kept in the across tissue matrix of presence/absence calls. An absent call was kept in the across tissue matrix of presence/absence calls for all inbred-gene combinations with only no or absent calls across tissues. These genes were designated in the following as ePAV which have an across tissue ePAV call of present and absent each for at least two inbreds (cf. Jin et al. [21]).

We used in analogy to Gabur et al. [30] the segregation of missing data in biparental populations to determine the percentage of ePAV that are due to gPAV. For the SNP from the SNParray dataset for which no missing data was observed, the *Q*_90_ of the major allele frequency was calculated per population to consider random deviations from an allele frequency of 0.5. For each population, each SNP was assigned to one of three categories based on the proportion of missing data: A: [0, *Q*_90_), B: [ *Q*_90_,1- *Q*_90_], C: (1- *Q*_90_, 1]. Category A to C can be interpreted as both parental inbreds have a present call, one parental inbred has a present and one an absent call, both parental inbreds have an absent call, respectively. A parental inbred was assigned an absent call at a SNP, if all populations derived from that parent were of category B or C. A parental inbred was assigned a present call at a SNP, if all populations derived from that parent were of category A or B. These 1,972 SNP that have a present and absent each for at least one inbred were designated in the following as gPAV-SNP (Additional file 1: Figure S2). A total of 14,843 barley genes comprised in their coding sequence one SNP from the SNParray and were designated in the following as genic SNP.

The 1,105 gPAV-SNP that were genic SNP and that were not within 30 bp of an insertion were designated as genic PAV-SNP.

The statistical power (1- *β*^∗^) to detect gPAV by mRNA sequencing was calculated as the percentage of genic PAV-SNP that were located within the coding sequence of ePAV. Furthermore, the empirical type I error (*α*^∗^) of our ePAV procedure was estimated as the proportion of genes that comprised a genic SNP, no genic PAV-SNP, but were detected as ePAV out of the total number of detected ePAV. In addition, we calculated the proportion of correct allele assignments (*o*) as the proportion of common presence/absence ePAV calls and presence/absence calls at genic PAV-SNP across all 23 inbreds. We estimated 1- *β*^∗^ and *α*^∗^ firstly for ePAV determined based on T of the entire gene as well as based on T calculated for 10 bp large windows surrounding the genic SNP.

A resampling procedure was used to determine the robustness of our ePAV detection procedure. For each gene, randomly 20% of the entire gene length were selected and transcript calling and ePAV detection were performed. This was repeated 50 times and the average of number and proportion of detected ePAV was calculated.The null hypothesis of a uniform distribution of ePAV across the genome and chromosomes was tested by a permutation procedure. The difference of mean gene length of ePAV and non-ePAV was tested for its statistical significance using a t-test. GO term enrichment analysis of ePAV was performed using the R-package topGO [51]. GO terms of newly annotated genes and newly identified genes were defined based on those available from the agriGO-database of homologous genes as described by Mascher et al. [6].

### Population genetic analyses

Variant calling was performed with samtools and bcftools. Sequence variants “SV” with mapping quality <55 were removed from the analysis. If the sequencing depth of a SV was smaller than 5, the allele call was set to “NA”. SV with a heterozygosity >10% were discarded and the alleles at remaining heterozygous sequence variants were set to “NA” for the corresponding inbreds. Biallelic sequence variants with a maximum of 20% missing information were retained. If the allele call was different between the tissues of the same inbred, the call with the higher sequencing depth was retained. Missing values in the matrices of SV and ePAV were mean imputed.

Associations among inbreds based on SV, ePAV, as well as T were revealed with a principal component analysis [52]. Pearson’s correlation coefficients were calculated between euclidean distance matrices of SV, ePAV, and T.Linkage disequilibrium measured as *r*^2^ [53] was calculated between ePAV and linked/unlinked SV.

### Genomic prediction

Each of the three phenotypic traits leaf angle, heading date, and plant height was analyzed across the four environments using mixed models. This allowed to estimate adjusted entry means as well as the heritability on an entry mean basis.The adjusted entry mean of each barley inbred for each trait was predicted using genomic best linear unbiased prediction (GBLUP) [54–57]. GBLUP was used as implemented in the R-package sommer [58], where only additive effects were modeled and the residuals were assumed to be normally distributed with mean 0 and variance $\sigma ^{2}_{e}$.The performance of the barley inbreds was predicted using different predictors: (i) SNParray, (ii) SV, (iii) ePAV, (iv) T _*l*_, (v) T _*s*_. *W* is a matrix of feature measurements for the respective predictors. The dimension of *W* is determined by the number of barley inbreds and the number of features in the corresponding predictor (*m*_*SNParray*_ = 44,045 *m*_*SV*_ = 133,566, *m*_*ePAV*_ = 38,810, *m*_*Tl*_ = 60,888, *m*_*Ts*_ = 67,844). The columns in *W* were centered and standardized to unit variance. For each predictor, an additive relationship matrix *G* was calculated according to VanRaden [59]. The matrices *G* of two or three predictors were weighted and summed up, resulting in one joined weighted relationship matrix [29]. A grid search, varying the relative weights in increments of 0.1, resulted in 66 different joined weighted relationship matrices. We calculated the prediction accuracy $[r(\hat {g},g)]$ for each examined scenario.The standard scheme for validation of genomic prediction was five-fold cross-validation. For this purpose, the 23 inbreds were randomly subdivided into five disjoint subsets. One subset was left out for validation, whereas the other four subsets were used as training set. This procedure was replicated 200 times, yielding a total of 1000 cross-validation runs. The median of the prediction accuracy across the 1,000 cross-validation runs was calculated. From the original data set of seedling samples, the number of reads was randomly reduced to 10, 5, 1, and 0.5% of the original number of reads per inbred. This procedure was replicated 30 times. For these subsets of reads, the above described work flow of read mapping, determination of gene expression, expression presence/absence variation, and sequence variant calling was performed. The prediction accuracy for the single predictors SV _*s*_, ePAV _*s*_, and T _*s*_ and the combination of these predictors, was calculated for leaf angle, heading date, and plant height as average across the 30 replications.

## Supplementary information

**Additional file 1** **Supplementary Table S1:** Summary of barley inbreds. **Supplementary Table S2:** Number of expression presence/absence variation (ePAV) observed for our detection procedure. **Supplementary Table S3:** Gene ontology term enrichment analysis. **Supplementary Fig. S1:** Characterization of the not annotated contigs established by the transcript calling. **Supplementary Fig. S2:** Procedure to evaluate the detection of presence/absence variation. **Supplementary Fig. S3:** Population structure of the 23 barley inbreds. **Supplementary Fig. S4:** Prediction accuracy of single predictors.

## Data Availability

The sequence data have been deposited in the NCBI Sequence Read Archive (SRA) under accession PRJNA534414. SV (SV_genome.csv), SNParray data (SNParray.csv), phenotypic data (aem_alltraitsDRRobs1718.csv), expression data (T_leaf.csv, T_seed-ling.csv), ePAV data (ePAV_consensus.csv, ePAV_leaf.csv, ePAV_seedling.csv) are contained within the paper and its additional files.

## Abbreviations

ePAV: Expression presence/absence variation
FPKM: Fragments per kilobase of exon model per million fragments mapped
GBLUP: Genomic best linear unbiased prediction
GO: Gene ontology
gPAV: Genomic presence/absence variation
IBSC: International Barley Sequencing Consortium
LD: Linkage disequilibrium
PAV: Presence/absence variation
PCA: Principal component analysis
SNP: Single nucleotide polymorphism
SV: Sequence variant
T: Transcriptomic variation
T _*a*_: Apex transcriptomic variation
T _*l*_: Leaf transcriptomic variation
T _*s*_: Seedling transcriptomic variation
