# Supplementary material for: Transcriptomic and presence/absence variation in the barley genome assessed from multi-tissue mRNA sequencing and their power to predict phenotypic traits
Source: BMC Genomics. 2019 Oct 29;20:787. doi: 10.1186/s12864-019-6174-3 (PMC6819542; doi:10.1186/s12864-019-6174-3)
Supplement: Supplementary file 1 — Additional file 1 Supplementary Table S1: Summary of barley inbreds. Supplementary Table S2: Number of expression presence/absence variation (ePAV) observed for our detection procedure. Supplementary Table S3: Gene ontology term enrichment analysis. Supplementary Fig. S1: Characterization of the not annotated contigs established by the transcript calling. Supplementary Fig. S2: Procedure to evaluate the detection of presence/absence variation. Supplementary Fig. S3: Population structure of the 23 barley inbreds. Supplementary Fig. S4: Prediction accuracy of single predictors. [file 12864_2019_6174_MOESM1_ESM.pdf]

SUPPLEMENTARY INFORMATION

Table S1: Inbred lines included in this study, their country of origin (CoO), row type, year of release, and the sequenced tissues.

					RNA sequencing		
Inbred name	BCC code	CoO	Row type	Year of release	Leaf	Seedling	Apex
HOR1842	HOR1842	AFG	6	1935	x	x	
HOR383	BCC1561	BGR	6	unknown	x	x	
Sanalta	BCC929	CAN	2	1930	[1]	[1]	
ItuNative	BCC502	CHN	6	unknown	x	x	
Sissy	BCC1413	GER	2	1990	x	x	x
Georgie	BCC1381	GBR	2	1975	x	x	
SprattArcher	BCC1415	GBR	2	1943	x	x	x
Lakhan	BCC533	IND	6	unknown	x	x	
Kharsila	HOR11403	IND	6	before 1911	x	x	
W23829/803911	HOR11374	ISR	2	unknown	x	x	x
Namhaebori	BCC667	KOR	6	unknown	x	x	
IG128216	BCC118	LBY	6	1983	x	[1]	
IG128104	BCC173	PAK	6	1974	x	x	
K10693	BCC1491	RUS	6	unknown	x	x	
IG31424	BCC190	SYR	2	1981	x	x	
HOR12830	HOR12830	SYR	6	unknown	x	x	
HOR7985	HOR7985	TUR	2	before 1969	x	x	x
K10877	BCC1503	TKM	6	unknown	x	x	x
HOR8160	HOR8160	TUR	2	before 1969	x	x	
Ancap2	BCC807	URY	6	1950	x	x	
CM67	BCC846	USA	6	1983	x	x	
Kombyne	BCC893	USA	6	1975	[1]	x	
Unumli-Arpa	BCC1470	UZB	2	unknown	x	x	x

[1] Samples were removed during the data cleaning process

Table S2: Number of expression presence/absence variation (ePAV) observed for our detection procedure.

Data set	#ePAV	#Genes	ePAV [%]
Barley, All	38,810	73,187	53.0
Barley, IBSC	28,340	60,162	47.1
Barley, newly annotated	9,286	11,523	80.6
Barley, newly identified	1,184	1,502	78.8

Table S3: The 15 GO terms of biological process that were most significantly enriched for ePAV.

GO.ID	Term	#Significant genes	#Expected genes	p-value
GO:0015074	DNA integration	761	166.95	$< 1e-30$
GO:0055114	oxidation-reduction process	705	241.69	$< 1e-30$
GO:0055085	transmembrane transport	251	119.07	$< 1e-30$
GO:0006278	RNA-dependent DNA biosynthetic process	337	142.98	$< 1e-30$
GO:0006605	protein targeting	105	48.82	$< 1e-30$
GO:0006508	proteolysis	630	433.82	$< 1e-30$
GO:0006468	protein phosphorylation	705	481.82	$< 1e-30$
GO:0006333	chromatin assembly or disassembly	170	99.18	$< 1e-30$
GO:0044238	primary metabolic process	4000	3255.6	$< 1e-30$
GO:0048544	recognition of pollen	77	19.28	1.1e-23
GO:0006281	DNA repair	144	99	1.3e-23
GO:0008152	metabolic process	5080	3967.22	4.9e-20
GO:0006313	transposition, DNA-mediated	46	8.42	7.1e-20
GO:0000723	telomere maintenance	60	18.54	1.6e-16
GO:0016358	dendrite development	20	8.78	1.1e-15

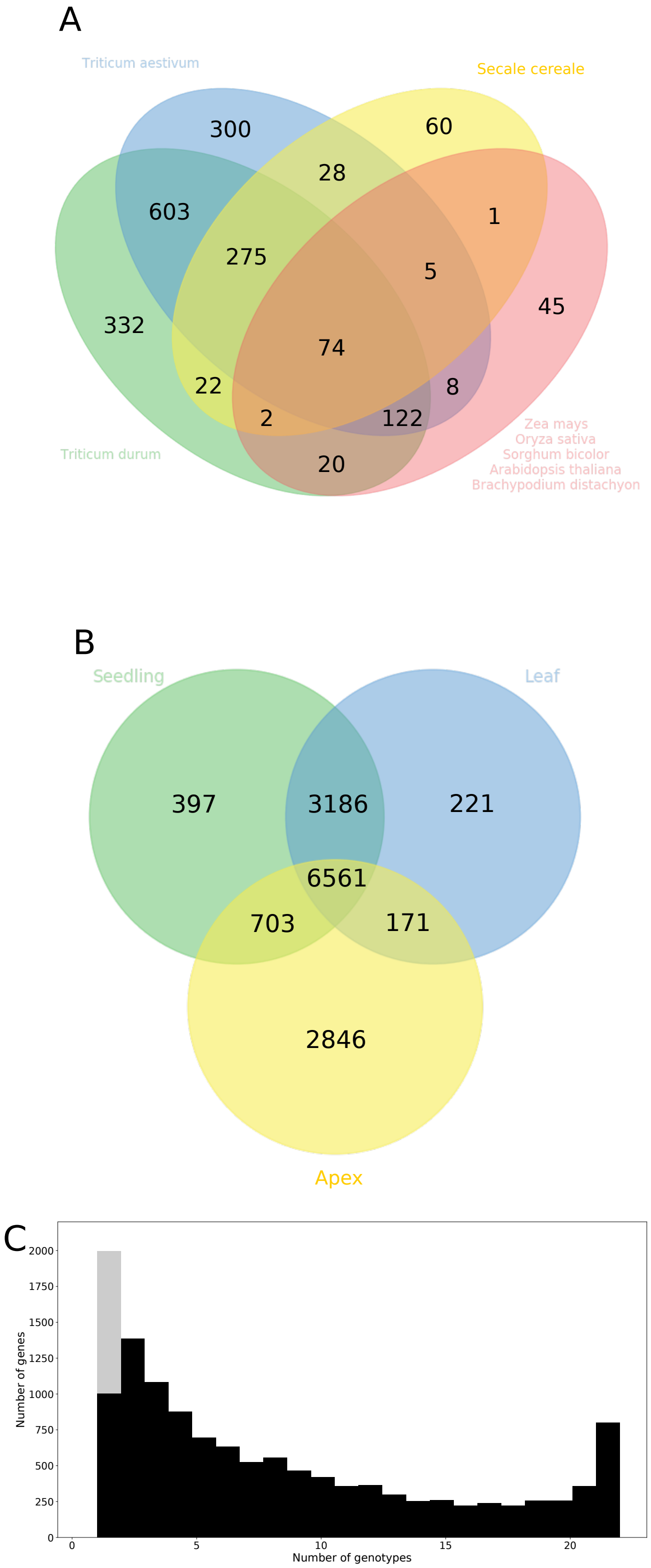

Fig. S1: Characterization of the not annotated contigs established by the transcript calling. A) Newly annotated genes which had based on BLASTn searches homology to eight different plant species (1,897). B) Expression of 11,523 newly annotated genes in the three different tissues. C) Number of inbred lines in which the not annotated contigs were called during the transcript calling. Gray bar shows the contigs detected in only one sample. The 11,523 genes, which were expressed in at least two samples, were marked in black and were designated as new annotated genes.

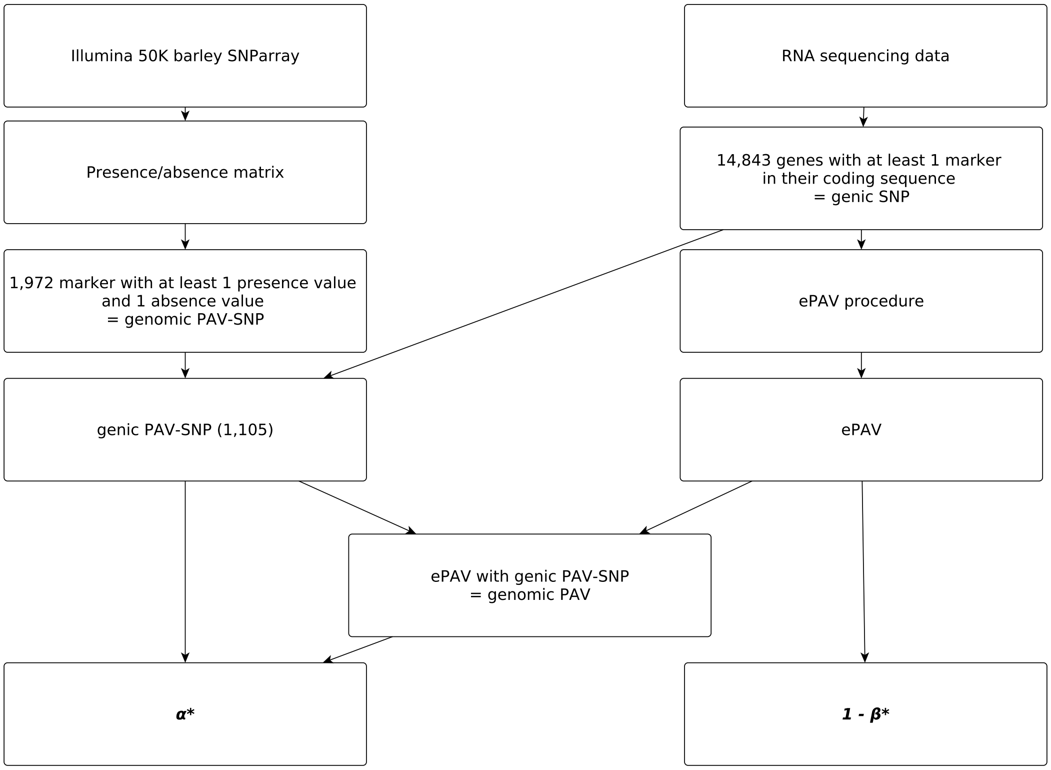

Fig. S2: Overview of the process to estimate the statistical power (1-β^*) and the empirical type I error rate (α^*) to detect genomic presence/absence variation (gPAV) by expression presence/absence variation (ePAV).

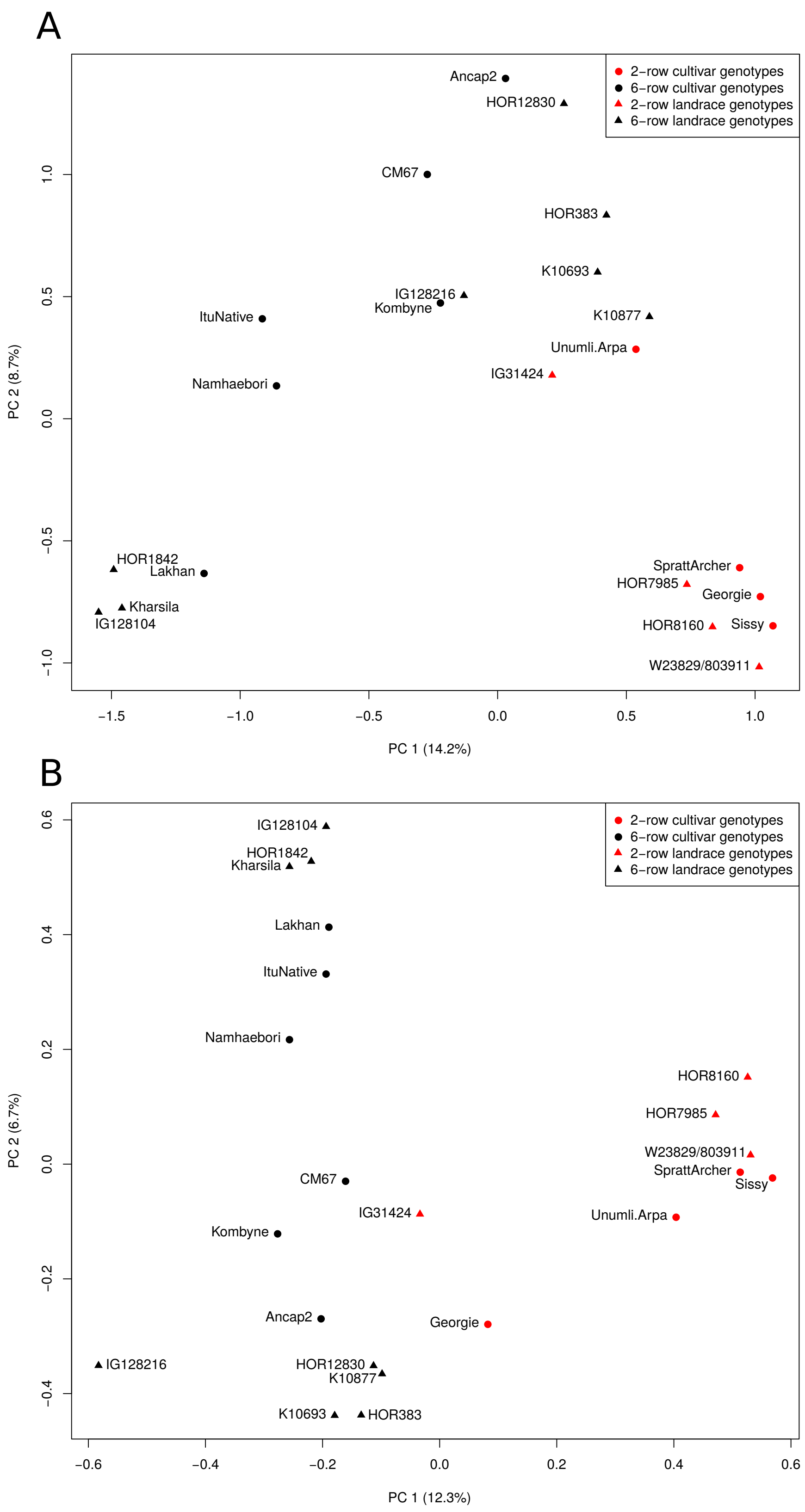

Fig. S3: Principal component analyses of the barley inbred lines considered in our study based on A) 133,566 genome-wide distributed sequence variants, and B) presence/absence allele call at 38,810 expression presence/absence variation. PC 1 and PC 2 are the first and second principal component, respectively, and number in parentheses refer to the proportion of variance explained by the principal components. Symbols identify landrace and cultivar inbreds and colors their row number.

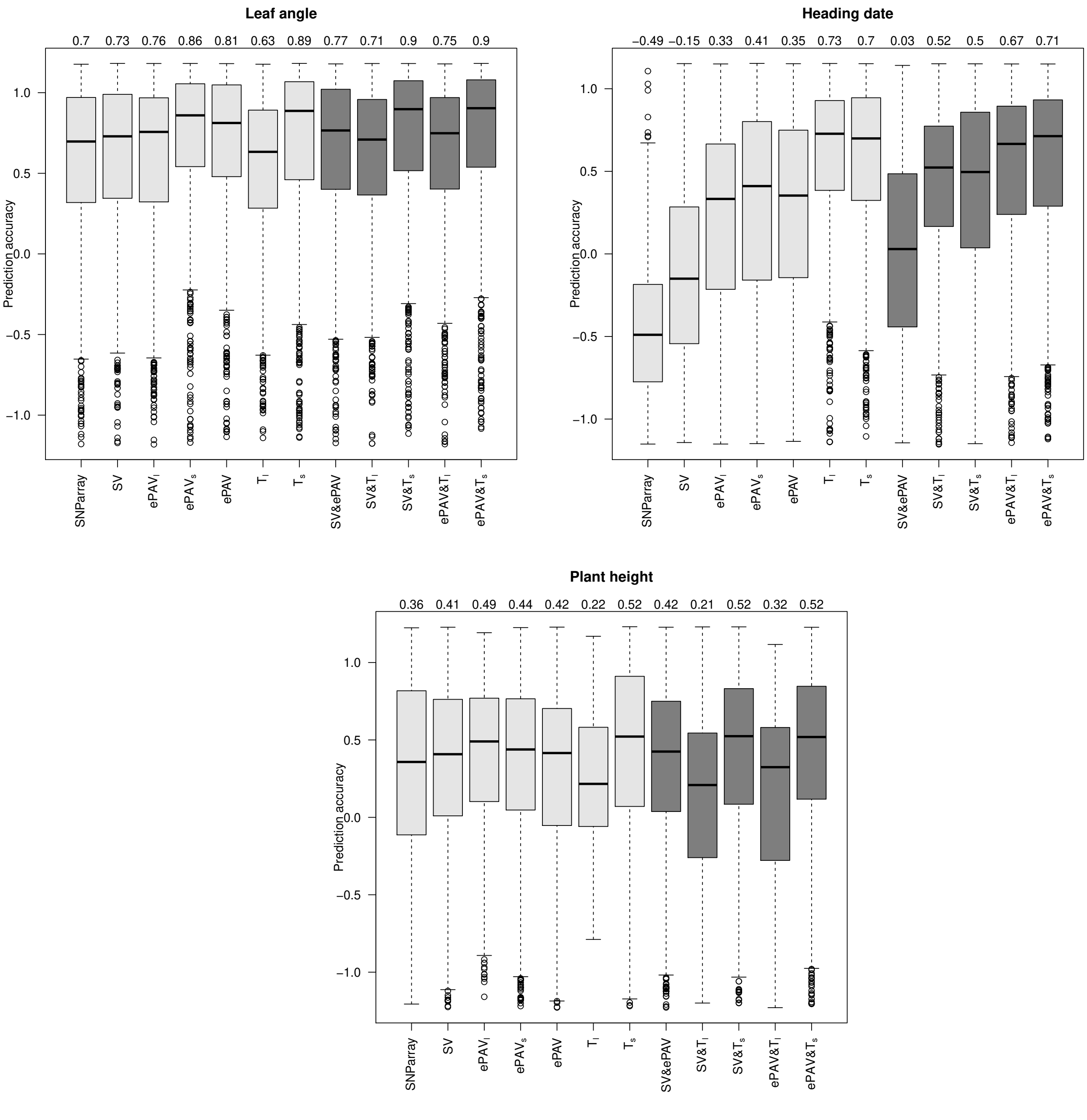

Fig. S4: Prediction accuracy for the barley inbreds of single predictors and combinations thereof for leaf angle, heading date, and plant height from 1,000 cross-validation runs with median prediction accuracy given above each column.
